# Type 1 Diabetes and Breast Cancer Risk: Evidence From an Updated Meta‐Analysis and Mendelian Randomization Study

**DOI:** 10.1155/ije/2837214

**Published:** 2026-11-05

**Authors:** Yi Xie, Qimou Lin, Jianhua Guan, Junda Li, Lin Yuan, Junhong Wen, Bo Xu

**Affiliations:** ^1^ Medical Education Center of Jinan University Jinan University Guangzhou China jnu.edu.cn; ^2^ Department of Breast Jiangmen Central Hospital Jiangmen China bjch.com; ^3^ Department of Thyroid and Breast Surgery Guangzhou First People’s Hospital Guangzhou China gzhosp.cn; ^4^ School of Medicine South China University of Technology Guangzhou China scut.edu.cn

**Keywords:** breast cancer, estrogen receptor–positive, Mendelian randomization, meta-analysis, observational study, Type 1 diabetes

## Abstract

**Background:**

It remains uncertain whether Type 1 diabetes (T1D) is linked to the risk of breast cancer (BC), with previous epidemiological findings yielding inconsistent conclusions.

**Methods:**

This study combined evidence from observational research through meta‐analysis and used a two‐sample Mendelian randomization (MR) framework to assess whether T1D contributes causally to BC risk. A total of five eligible articles encompassing nine cohort studies were included in the meta‐analysis. Genetic variants with strong associations to T1D were sourced from the IEU Open GWAS database (GWAS ID: ebi‐a‐GCST90018925). Summary‐level data for BC and its estrogen receptor (ER) subtypes were obtained from the FinnGen consortium (release R12).

**Results:**

The pooled meta‐analysis showed a statistically significant inverse association between T1D and BC risk (pooled relative effect estimate = 0.91, 95% CI: 0.85–0.98, *p* = 0.01). MR analyses provided evidence consistent with an inverse association between genetically predicted T1D and overall BC risk (IVW HR = 0.96, 95% CI: 0.92–1.00, *p* = 0.033), with a similar inverse association observed for ER‐positive BC (IVW HR = 0.95, 95% CI: 0.92–0.99, *p* = 0.013), whereas no statistically significant association was observed for ER‐negative BC. Sensitivity analyses indicated stable effect estimates despite evidence of horizontal pleiotropy detected by MR‐PRESSO, while the MR‐Egger intercept showed no evidence of directional pleiotropy.

**Conclusion:**

The combined observational and genetic evidence is consistent with a possible inverse association between T1D and BC risk, particularly for ER‐positive disease, whereas no significant association was observed for ER‐negative BC. Further studies are warranted to confirm these findings and clarify the underlying mechanisms.

## 1. Introduction

Type 1 diabetes (T1D), which constitutes about 5%–10% of all diabetes cases globally, is a major chronic metabolic condition [1, 2] Epidemiological surveys indicate that nearly 63% of individuals with T1D are diagnosed before the age of 30, and the median onset occurs around 24 years [3]. T1D currently affects approximately 8.4 million people worldwide [2]. Its prevalence has been increasing steadily at an annual rate of approximately 2%–3%, although considerable geographic heterogeneity exists, likely reflecting variations in genetic predisposition and environmental determinant [4, 5]. Given these trends, the global population living with T1D is projected to rise markedly over the coming decades, reaching an estimated 13.5–17.4 million individuals by 2040 [2, 4]. This rapid rise in prevalence is expected to have profound implications for individuals, healthcare systems, and economies worldwide [6].

Among females, breast cancer (BC) is the most frequently diagnosed malignancy, ranking first in incidence across 157 countries and serving as the predominant contributor to cancer mortality in 112 countries [7–9]. Extensive observational evidence has sought to clarify whether T1D influences BC risk; nonetheless, the current literature provides conflicting and inconclusive results. As early as 1997, Hjalgrim et al. reported no statistically significant association between T1D and BC risk, with similar conclusions drawn by Shu et al., Hsu et al., and Swerdlow et al. in later studies [10–13]. In contrast, Carstensen et al.’s extensive multinational cohort study, spanning five countries, suggested a potential protective effect of T1D against BC, whereas Xiong et al. reported an increased risk in women with T1D compared to nondiabetic controls [14, 15]. These inconsistent findings may reflect differences in the definition of T1D, age at diabetes onset, follow‐up duration, adjustment for reproductive and metabolic factors, insulin treatment patterns, and the demographic characteristics of the study populations [12, 14]. In addition, most previous studies evaluated overall BC without considering hormone receptor–defined subtypes. Therefore, both an updated synthesis of observational evidence and a complementary genetic analysis are needed to clarify the direction and potential causality of the association.

The potential relationship between T1D and BC may involve several distinct biological pathways. Unlike Type 2 diabetes (T2D), which is commonly characterized by insulin resistance and compensatory hyperinsulinemia, T1D is an autoimmune disease characterized by progressive destruction of pancreatic β‐cells and insulin deficiency [16, 17]. Immune dysregulation and inflammatory processes are central features of T1D pathogenesis [16]. In addition, the relative portal hypoinsulinemia associated with subcutaneous insulin administration in T1D may influence hepatic endocrine function, including sex hormone‐binding globulin (SHBG) production [18]. Alterations in the growth hormone (GH)/insulin‐like growth factor axis, particularly reduced circulating insulin‐like growth factor 1 (IGF‐1) levels, have also been reported in individuals with T1D [19, 20]. These metabolic and endocrine alterations may be relevant to BC because insulin and IGF signaling can activate phosphoinositide 3‐kinase (PI3K)/protein kinase B (AKT)/mechanistic target of rapamycin (mTOR) and mitogen‐activated protein kinase (MAPK) pathways involved in mammary epithelial proliferation, survival, and tumor progression [21, 22]. However, these mechanisms remain hypothetical in the context of the observed T1D–BC association and require further investigation. Given these inconsistencies and the potential biological mechanisms linking T1D and BC, further investigation is warranted. Therefore, by pooling data from prior observational studies, this study offers a comprehensive meta‐analytic evaluation of the T1D–BC association. Complementing this, a Mendelian randomization (MR) approach was applied using T1D‐associated genetic variants as instrumental variables to assess whether the observed association was consistent with a potential causal relationship while reducing, although not eliminating, the influence of residual confounding and reverse causation [23, 24]. By integrating both meta‐analytic and MR approaches, this study strives to present robust and multifaceted insights into the T1D–BC relationship, yielding new understanding of potential causal pathways and implications for clinical practice.

## 2. Methods

### 2.1. Meta‐Analysis

#### 2.1.1. Study Selection

The protocol for this systematic review and meta‐analysis was not prospectively registered in PROSPERO or another systematic review registry. An extensive database search encompassing PubMed, Embase, Web of Science, and the Cochrane Library was performed to retrieve English‐language studies released from January 1, 2000, through March 23, 2026. Grey literature databases, conference abstracts, trial registries, dissertations, and unpublished studies were not systematically searched. Only full‐text articles published in peer‐reviewed journals were considered eligible.

Both Medical Subject Headings (MeSH) and free‐text terms covering T1D, BC, and related risk determinants were utilized in constructing the search strategy. The detailed search terms were as follows: (“Diabetes Mellitus, Type 1” OR “type 1 diabetes” OR “type I diabetes” OR “insulin dependent diabetes” OR “insulin‐dependent diabetes mellitus” OR “juvenile diabetes” OR “T1D”) AND (“Breast Neoplasms” OR “breast cancer” OR “breast neoplasm” OR “breast carcinoma” OR “mammary carcinoma” OR “breast tumor” OR “breast tumour” OR “mammary neoplasm”) AND (“risk” OR “relative risk” OR “RR” OR “odds ratio” OR “OR” OR “hazard ratio” OR “HR” OR “standardized incidence ratio” OR “SIR” OR “incidence rate” OR “incidence” OR “risk ratio” OR “rate ratio”).

We included observational studies such as cohort or case–control studies examining the association between T1D and the incidence or risk of BC, reporting effect estimates such as relative risk (RR), odds ratio (OR), standardized incidence ratio (SIR), or hazard ratio (HR) with corresponding 95% confidence intervals (CIs). When duplicate or overlapping data were identified across studies, only the study providing the most complete or the earliest information was retained.

#### 2.1.2. Data Extraction

Using a predesigned standardized form, two independent researchers (Yi Xie and Jianhua Guan) retrieved relevant information from each eligible study. Data collection covered: first author, year of publication, country, study design, definition of T1D, population of T1D, BC cases among T1D patients, person‐years at risk, follow‐up time (years), and reported effect sizes (RR, OR, HR, or SIR) with corresponding 95% CIs. When any discrepancies appeared, another reviewer (Qimou Lin) participated in the discussions, and we negotiated to resolve the disputes until a consensus was achieved.

#### 2.1.3. Quality Assessment

The methodological rigor of each observational study was appraised according to the Newcastle–Ottawa Scale (NOS) [25]. Studies with NOS scores of ≥ 5 were considered to be of acceptable methodological quality.

#### 2.1.4. Statistical Analysis

The primary outcome was the association between T1D and the incidence of BC, summarized as a pooled relative effect estimate with a corresponding 95% CI. Although the eligibility criteria permitted studies reporting HRs, RRs, ORs, or SIRs, the studies included in the final quantitative synthesis reported only HRs or SIRs. Given that BC was a relatively uncommon outcome in the included populations and that both HRs and SIRs represent ratio measures of relative occurrence, these estimates were synthesized as measures of a common underlying relative association. However, because HRs and SIRs are not mathematically or conceptually equivalent, the pooled estimate was interpreted cautiously as a summary relative effect rather than as a homogeneous HR [26, 27]. The reported estimates were logarithmically transformed and pooled using the generic inverse‐variance method, with each study weighted according to the inverse of the variance of its effect estimate [26]. A random‐effects model was applied to account for anticipated clinical and methodological heterogeneity among studies [26]. We evaluated statistical heterogeneity using the *I* ^2^ statistic, where heterogeneity was categorized as low, moderate, or high when the *I* ^2^ statistic reached approximately 25%, 50%, and 75%, respectively [28]. With fewer than 10 studies eligible for analysis, potential publication bias was explored using contour‐enhanced funnel plots alongside the trim‐and‐fill technique to evaluate the impact of unreported studies [29]. To examine result reliability, sensitivity analyses were undertaken, with all computations executed in R (v4.3.1) utilizing the “meta” package (v8.0‐1).

### 2.2. MR

#### 2.2.1. Study Design

The MR analysis is built upon three essential assumptions: single‐nucleotide polymorphisms (SNPs) must have a robust association with T1D (the “relevance” criterion); they must be unrelated to confounding factors (the “independence” criterion); and their influence on BC must be mediated exclusively via T1D (the “exclusion restriction” criterion) [30]. Further details are provided in Figure 1.

**FIGURE 1:**
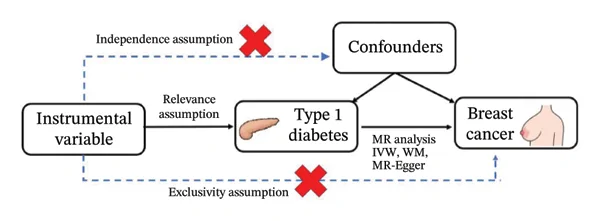
Mendelian randomization flowchart for T1D and BC.

#### 2.2.2. Data Source

The study population of the original GWAS comprised individuals of European descent. Exposure data for T1D were obtained from the IEU Open GWAS database (GWAS ID: ebi‐a‐GCST90018925), which was selected based on its large sample size, recent availability, and greater number of genetic variants available for instrument selection [31]. Outcome data for overall BC, estrogen receptor (ER)–positive BC, and ER‐negative BC were derived from FinnGen (R12), an independent cohort chosen to minimize potential sample overlap with the exposure dataset. Detailed characteristics of all GWAS datasets are presented in Table S1.

#### 2.2.3. Selection of Instrumental Variables

At the preliminary stage, we extracted SNPs that exhibited genome‐wide significant associations (*p* < 5 × 10^−8^) with the exposure trait. Linkage disequilibrium clumping (*R* ^2^ < 0.001; distance > 10 Mb) was subsequently performed to ensure SNP independence [32]. To ensure independence, we screened LDlink (https://ldlink.nh.gov) for potential pleiotropic confounders. The screening focused on established or plausible BC risk factors, including body mass index and other adiposity‐related traits, smoking, alcohol consumption, reproductive traits, age at menarche and menopause, parity, and relevant hormonal traits. LDtrait was initially queried using a broad linkage disequilibrium threshold (*R* ^2^ ≥ 0.1 within ±500 kb) to maximize the sensitivity of phenotype‐association screening. Returned phenotype associations were evaluated according to the strength of LD, statistical evidence, and their biological relevance to established BC risk factors; variants were not excluded solely because a phenotype association was identified for a weakly linked neighboring variant. SNPs absent in the outcomes dataset and palindrome SNPs were excluded to mitigate bias. *F*‐statistics were calculated to assess instrument strength, with values > 10 considered indicative of sufficiently strong instruments and a low likelihood of substantial weak‐instrument bias [32].

#### 2.2.4. Statistical Methods

Three MR approaches—inverse variance weighted (IVW), weighted median, and MR‐Egger—were applied to quantify causal associations [33]. Given its effectiveness in assessing causal relationships, our analysis predominantly hinges on the IVW method. HR was utilized to quantify the influence of T1D on BC. The IVW method was prespecified as the primary analysis, while weighted‐median and MR‐Egger estimates were considered complementary sensitivity analyses. The overall interpretation was based on the direction, magnitude, and precision of the effect estimates together with the results of heterogeneity and pleiotropy assessments. Heterogeneity among genetic instruments was evaluated using Cochran’s *Q* test, where *p* values exceeding 0.05 suggested its absence. Horizontal pleiotropy was investigated through MR‐Egger regression and MR‐PRESSO, with a zero intercept (*p* > 0.05) indicating no evidence of pleiotropy [34, 35]. R software (version 4.3.1) was used to perform all statistical analyses, utilizing the packages “TwoSampleMR” (0.6.3), “MRPRESSO” (1.0), and “MendelianRandomization” (0.3.2).

## 3. Results

### 3.1. Meta‐Analysis

#### 3.1.1. Systematic Literature Search

In total, 1079 records were identified from four databases: PubMed (99), EMBASE (693), Web of Science (221), and the Cochrane Library (66). After eliminating 178 duplicates, 901 articles were available for screening. Title and abstract review led to the exclusion of 812 studies based on predefined criteria. After full‐text assessment of 89 articles, 84 were removed based on the exclusion criteria described below: insufficient data (*n* = 43), review articles (*n* = 17), irrelevance to the research topic (*n* = 21), or potential overlap with the same study population (*n* = 3) [36–38]. Ultimately, nine studies derived from five articles were included in the final quantitative synthesis [10–12, 14, 15]. Further details are provided in Figure 2.

**FIGURE 2:**
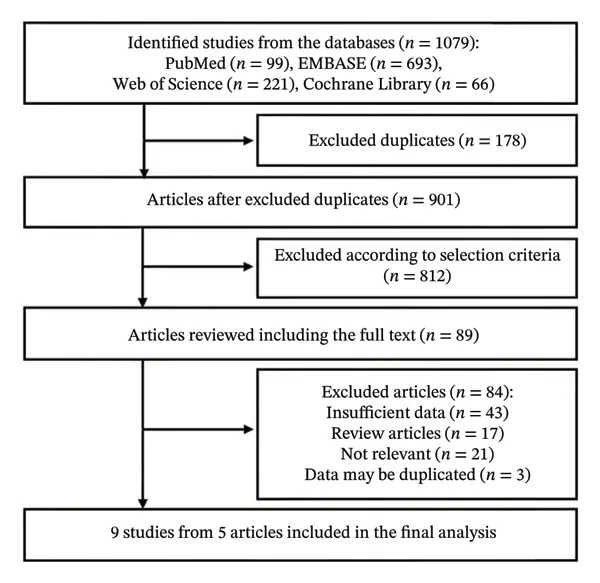
Flow diagram of identification of relevant studies.

#### 3.1.2. Study Description and Quality Assessment

A total of five publications comprising nine independent study populations were considered eligible and incorporated into the current meta‐analysis. Eight study populations originated from European cohorts and one from an Asian cohort. Of these, eight employed a population‐based retrospective cohort design, and one was conducted prospectively. The diagnosis of T1D was primarily determined using International Classification of Diseases (ICD) codes, version 9 or 10, or based on age at diagnosis to differentiate T1D from T2D. The enrollment periods spanned from 1964 to 2012. Eight studies reported person‐years at risk, and four provided the median (mean) year of follow‐up. Regarding outcome assessment, six study populations reported HR and three reported SIR. The methodological quality of the included studies was assessed using the NOS, with total scores ranging from 6 to 8, indicating overall moderate to high quality. Domain‐level NOS assessment showed that the principal methodological limitations were incomplete adjustment for BC‐specific confounders and potential misclassification of T1D in studies using age at diabetes diagnosis as a proxy for diabetes type (Table S2). Outcome ascertainment and follow‐up were generally robust across the included studies. Detailed characteristics of the included studies, outcome measures, and quality evaluations are presented in Table 1.

**TABLE 1:** General characteristics of the studies included in the final analysis.

Study	Year	Country	Type of study	Definition of T1D	Years enrolled	Population of T1D	BC cases among T1D patients	Person‐years at risk	Follow‐up time (years)	SIR/HR (95% CI)	NOS score
Shu et al. [10]	2010	Sweden	Retrospective cohort study	age < 21	1964–2006	11,290	35	210,712	Median (17), mean (18)	SIR: 0.87 (0.61–1.21)	7
Hsu et al. [11]	2015	Taiwan	Retrospective cohort	ICD 9	2000–2007	7752	56	47,741	Mean (6)	SIR: 1.04 (0.78–134)	6
Carstensen et al. [14]	2016	Denmark	Retrospective cohort	age < 40	1995–2012	NA	184	293,900	NA	HR: 0.74 (0.64–0.86)	6
Carstensen et al. [14]	2016	Finland	Retrospective cohort	age < 40	1972–2010	NA	546	648,000	NA	HR: 0.88 (0.81–0.96)	6
Carstensen et al. [14]	2016	Sweden	Retrospective cohort	age < 40	1987–2011	NA	710	146,800	NA	HR: 0.95 (0.89–1.03)	6
Carstensen et al. [14]	2016	Scotland	Retrospective cohort	age < 40	1995–2011	NA	99	645,200	NA	HR: 0.91 (0.75–1.11)	6
Carstensen et al. [14]	2016	Australia	Retrospective cohort	age < 40	2000–2008	NA	184	255,400	NA	HR: 0.9 (0.79–1.03)	6
Swerdlow et al. [12]	2023	UK	Retrospective cohort	age < 30	1972–1993	10,935	212	NA	Mean (30)	SIR: 0.96 (0.83–1.09)	6
Xiong et al. [15]	2023	UK	Prospective cohort	ICD 10	2006–2010	575	26	6075	Median (11)	HR: 1.52 (1.03–2.23)	7

*Note:* ICD‐9, International Classification of Disease ninth Version; ICD‐10, International Classification of Disease tenth Version.

Abbreviations: BC, breast cancer; CI, confidence interval; HR, hazard ratio; NA, not available; NOS, Newcastle–Ottawa scale; SIR, standardized incidence ratio; T1D, Type 1 diabetes.

#### 3.1.3. Meta‐Analysis

The pooled analysis showed a statistically significant inverse association between T1D and BC risk (pooled relative effect estimate = 0.91, 95% CI: 0.85–0.98, *p* = 0.01), although moderate between‐study heterogeneity was observed (*I* ^2^ = 54%, *p* = 0.03). Detailed study‐specific results are presented in Figure 3.

**FIGURE 3:**
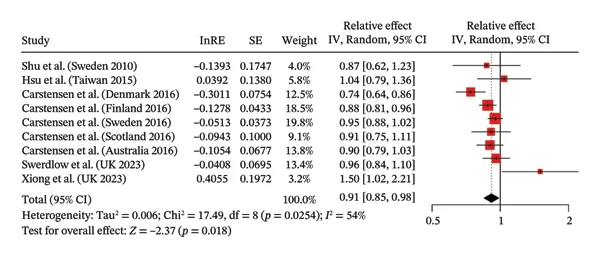
Forest plot of the association between T1D and BC risk.

Subgroup analyses were performed according to effect measure, definition of T1D, publication source/population of T1D, number of BC cases among patients with T1D, and person‐years at risk (Table 2). The inverse association between T1D and BC risk remained statistically significant across several subgroup analyses, with a pooled relative effect estimate of 0.90 (95% CI: 0.82–0.99; *p* = 0.04) in the effect‐measure subgroup analysis, 0.89 (95% CI: 0.84–0.95; *p* < 0.01) in studies using an age‐based definition of T1D, 0.88 (95% CI: 0.81–0.95; *p* < 0.01) in studies from Carstensen et al., in which the T1D population size was not reported, 0.89 (95% CI: 0.82–0.96; *p* < 0.01) in studies with more than 100 BC cases among patients with T1D, and 0.86 (95% CI: 0.79–0.93; *p* < 0.01) in studies contributing more than 200,000 person‐years at risk.

**TABLE 2:** Subgroup analyses of the association between Type 1 diabetes and breast cancer risk.

Subgroup	No. of cohorts	Pooled effect (95% CI)	*p* value	*I* ^2^ (%)	*P* for subgroup difference
Overall	9	0.91 (0.85–0.98)	0.011	54.0	
Effect measure					0.430
HR	6	0.90 (0.82–0.99)	0.038	69.2	
SIR	3	0.96 (0.86–1.08)	0.522	0.0	
Definition of T1D					0.027
Age‐based	7	0.89 (0.84–0.95)	0.001	44.0	
ICD‐based	2	1.22 (0.85–1.73)	0.281	56.8	
Publication source/population of T1D					0.086
Carstensen study/not reported	5	0.88 (0.81–0.95)	0.002	57.8	
Other studies/reported	4	1.02 (0.88–1.18)	0.805	28.9	
BC cases among T1D patients					0.179
< 100	4	1.02 (0.84–1.24)	0.847	45.8	
> 100	5	0.89 (0.82–0.96)	0.004	61.8	
Person‐years at risk					0.050
< 200,000	4	1.01 (0.87–1.18)	0.887	41.2	
> 200,000	4	0.86 (0.79–0.93)	< 0.001	39.7	

*Note:* Random‐effects models were used for all analyses. Differences between subgroups were assessed using tests for subgroup differences. One study was excluded from the person‐years subgroup analysis because person‐years at risk were unavailable.

Abbreviations: BC, breast cancer; CI, confidence interval; HR, hazard ratio; ICD, International Classification of Diseases; SIR, standardized incidence ratio; T1D, Type 1 diabetes.

A significant difference was observed between subgroups according to the definition of T1D (P for subgroup difference = 0.03). In contrast, no significant subgroup differences were observed according to effect measure (*p* = 0.43), publication source/population of T1D reporting (*p* = 0.09), or number of BC cases among patients with T1D (*p* = 0.18). The difference according to person‐years at risk was borderline significant (*p* = 0.05). These subgroup findings should be interpreted cautiously because of the limited number of cohorts within some subgroups.

#### 3.1.4. Assessment of Publication Bias

Visual inspection of the contour‐enhanced funnel plot and trim‐and‐fill analysis did not suggest substantial asymmetry; however, these findings should be interpreted cautiously because fewer than 10 study populations were available. Detailed study‐specific results are presented in Figures 4 and 5.

**FIGURE 4:**
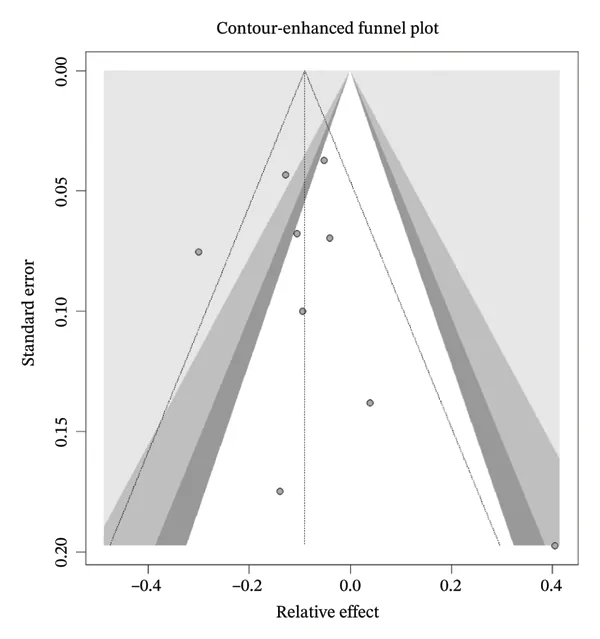
Contour‐enhanced funnel plot of the association between T1D and BC risk.

**FIGURE 5:**
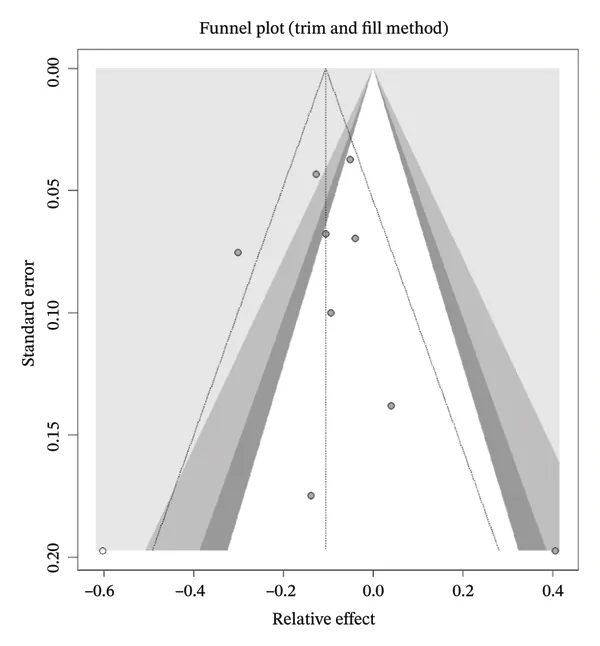
Trim‐and‐fill funnel plot of the association between T1D and BC risk.

#### 3.1.5. Sensitivity Analyses

As depicted in Figure 6, the sensitivity analysis confirmed the stability of the pooled outcomes, which remained consistent after sequential exclusion of each study.

**FIGURE 6:**
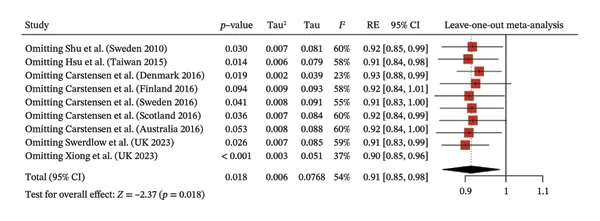
Leave‐one‐out sensitivity analysis of the association between T1D and BC risk.

### 3.2. MR

#### 3.2.1. Screening of SNPs

A total of 18 independent SNPs strongly associated with T1D were initially identified as candidate instrumental variables. Among these, rs3842753 was excluded because of its potential association with BMI, leaving 17 SNPs for the final MR analyses. The F‐statistics of the retained instruments ranged from 30.10 to 1402.98, with a mean F‐statistic of 177.26. All F‐statistics were substantially above the conventional threshold of 10, indicating that weak‐instrument bias was unlikely to materially affect the MR estimates. Detailed characteristics and strength of the genetic instrumental variables are presented in Supporting Table S3.

#### 3.2.2. Relationship Between T1D and BC Risk

The IVW and weighted‐median analyses indicated an inverse association between genetically predicted T1D and overall BC risk (IVW: HR = 0.957, 95% CI: 0.919–0.997, *p* = 0.033; weighted median: HR = 0.959, 95% CI: 0.935–0.983, *p* = 0.001). However, the MR‐Egger analysis yielded more conservative and statistically nonsignificant results (HR = 0.979, 95% CI: 0.915–1.047, *p* = 0.542). Detailed study‐specific results are presented in Figure 7, Table 3, and Table S4.

**FIGURE 7:**
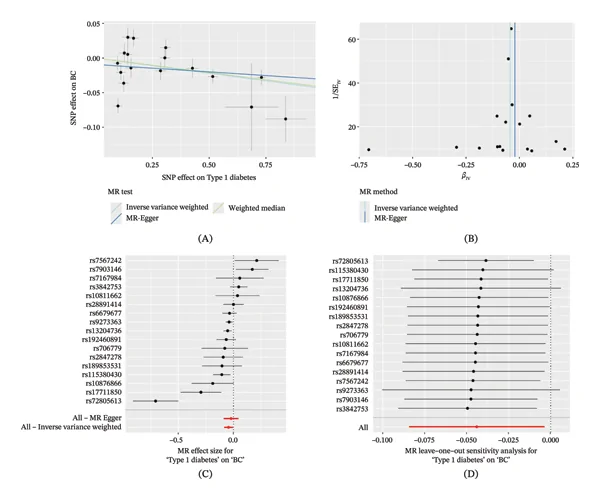
Mendelian randomization analysis of the association between T1D and BC risk. (A) Scatter plot. (B) Funnel plot. (C) Forest plot. (D) Leave‐one‐out sensitivity analysis.

**TABLE 3:** MR of the correlation between T1D and BC.

Outcome	Used SNPs	Method	Beta	se	HR (95% CI)	*P*	Heterogeneity test		Pleiotropy test	
							*Q*	*Q*_pval	Egger intercept *p* value	MR‐PRESSO Global test *p* value
Breast cancer	17	MR‐Egger	−0.021	0.034	0.979 (0.915–1.047)	0.542	71.481	< 0.001	0.422	< 0.001
	17	IVW	−0.044	0.021	0.957 (0.919–0.997)	0.033	74.728	< 0.001		
	17	Weighted median	−0.042	0.013	0.959 (0.935–0.983)	0.001				

ER‐positive BC	17	MR‐Egger	−0.029	0.032	0.971 (0.912–1.034)	0.377	40.538	< 0.001	0.476	0.001
	17	IVW	−0.048	0.019	0.953 (0.918–0.990)	0.013	41.980	< 0.001		
	17	Weighted median	−0.049	0.015	0.952 (0.925–0.981)	0.001				

ER‐negative BC	17	MR‐Egger	−0.003	0.040	0.997 (0.921–1.078)	0.934	43.913	< 0.001	0.352	0.001
	17	IVW	−0.034	0.024	0.967 (0.921–1.014)	0.162	46.620	< 0.001		
	17	Weighted median	−0.026	0.180	0.974 (0.940–1.010)	0.147				

Heterogeneity tests revealed significant variability among IVs (Cochran’s *Q* for IVW = 74.728, *p* < 0.001; MR‐Egger = 71.481, *p* < 0.001). MR‐PRESSO analysis identified significant horizontal pleiotropy (Global Test *p* < 0.001). After excluding outlier SNPs, the causal estimate strengthened (outlier‐corrected causal estimate = −0.038; SE = 0.014; *p* = 0.018) without evidence of distortion (distortion Test *p* = 0.517). Based on the Egger regression intercept (−0.009; SE = 0.011; *p* = 0.422), there was no statistical evidence of horizontal pleiotropy affecting the results. Leave‐one‐out sensitivity analyses verified the stability of the results, with no single SNP exerting a notable impact on the outcomes. Detailed study‐specific results are presented in Figure 7 and Table 3.

The consistency of findings from IVW and weighted median analyses, alongside a similar trend in MR‐Egger, provides justification for considering the IVW approach as the principal reference analysis. Collectively, these findings provide genetic evidence consistent with a possible inverse association between T1D and overall BC risk, in line with the results of the meta‐analysis.

#### 3.2.3. Relationship Between T1D and ER‐Positive BC Risk

The IVW and weighted median analyses consistently demonstrated that T1D was significantly associated with a lower risk of ER‐positive BC (IVW: HR = 0.953, 95% CI: 0.918–0.990, *p* = 0.013; weighted median: HR = 0.952, 95% CI: 0.925–0.981, *p* = 0.001). In contrast, MR‐Egger analysis yielded more conservative and nonsignificant results (HR = 0.971, 95% CI: 0.912–1.034, *p* = 0.377). Detailed study‐specific results are presented in Figure 8, Table 3, and Table S5.

**FIGURE 8:**
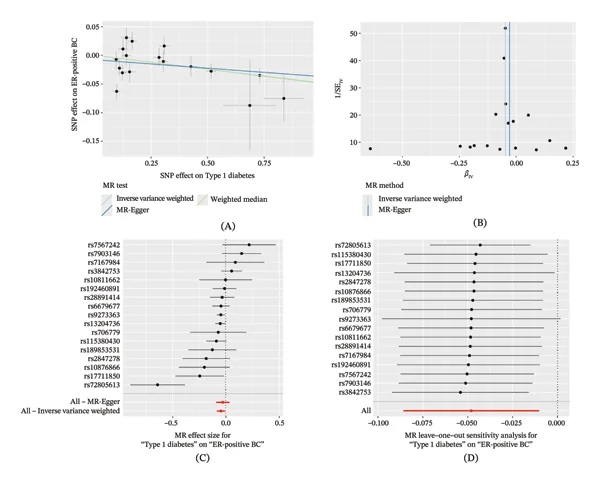
Mendelian randomization analysis of the association between T1D and ER‐positive BC risk. (A) Scatter plot. (B) Funnel plot. (C) Forest plot. (D) Leave‐one‐out sensitivity analysis.

Heterogeneity testing demonstrated statistically significant differences (Cochran’s *Q* for IVW = 41.980, *p* < 0.001; MR‐Egger = 40.538, *p* < 0.001). MR‐PRESSO analysis identified significant horizontal pleiotropy (Global Test *p* = 0.001). After excluding outlier SNPs, the causal estimate became more pronounced (outlier‐corrected causal estimate = −0.043; SE = 0.014; *p* = 0.009), with results remaining consistent pre‐ and post‐correction (distortion test *p* = 0.516). Egger’s regression intercept (Egger intercept = −0.008; SE = 0.011; *p* = 0.476) indicated no significant pleiotropy. Robustness of the findings was additionally supported by the leave‐one‐out analysis. Detailed study‐specific results are presented in Figure 8 and Table 3.

These findings provide evidence consistent with an inverse association between genetically predicted T1D and ER‐positive BC risk.

#### 3.2.4. Relationship Between T1D and ER‐Negative BC Risk

No statistically significant association was observed between genetically predicted T1D and ER‐negative BC risk across all methods (IVW: HR = 0.967, 95% CI: 0.921–1.014, *p* = 0.162; MR‐Egger: HR = 0.997, 95% CI: 0.921–1.078, *p* = 0.934; weighted median: HR = 0.974, 95% CI: 0.940–1.010, *p* = 0.147). Detailed study‐specific results are presented in Figure 9 and Table 3.

**FIGURE 9:**
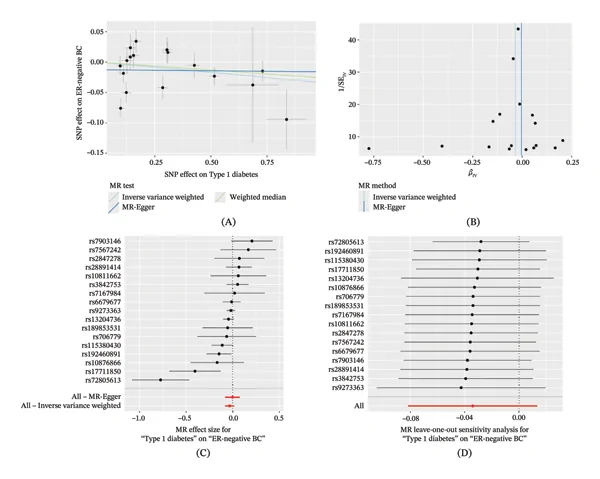
Mendelian randomization analysis of the association between T1D and ER‐negative BC risk. (A) Scatter plot. (B) Funnel plot. (C) Forest plot. (D) Leave‐one‐out sensitivity analysis.

Heterogeneity tests revealed significant variability among the instrumental variables (Cochran’s *Q* for IVW = 46.620, *p* < 0.001; MR‐Egger = 43.913, *p* < 0.001), indicating potential heterogeneity across SNPs. Significant horizontal pleiotropy was identified by the MR‐PRESSO global test (*p* = 0.001), suggesting potential exposure‐independent effects of several instrumental variables. After excluding outlier SNPs, the causal estimate was −0.028 (SE = 0.018; *p* = 0.146), showing a consistent but statistically nonsignificant association. The distortion test (*p* = 0.576) indicated that outlier removal did not materially alter the causal estimate, supporting the robustness of the findings. No directional pleiotropy was detected based on the MR‐Egger intercept test (intercept = −0.013; SE = 0.013; *p* = 0.352), implying that overall bias due to pleiotropy was minimal. Leave‐one‐out tests conducted as part of sensitivity analyses further validated the consistency of the findings. Detailed study‐specific results are presented in Figure 9, Table 3, and Table S6.

Overall, the MR analyses provided no statistically significant evidence of an association between genetically predicted T1D and ER‐negative BC risk.

## 4. Discussion

This study provided an in‐depth assessment of the link between T1D and BC risk by integrating evidence from observational studies using a meta‐analytic approach and incorporating MR analyses. The meta‐analysis showed a statistically significant inverse association between T1D and BC risk, although moderate heterogeneity was observed across studies and moderate heterogeneity was observed across studies. Complementary MR analyses provided genetic evidence consistent with an inverse association with overall and ER‐positive BC risk, whereas no significant association was observed for ER‐negative BC. The consistency of these findings across the observational and genetic analyses provides convergent evidence supporting a possible inverse association between T1D and overall and ER‐positive BC risk.

Recent meta‐analyses have largely focused on evaluating the association of T2D or diabetes as a whole with the risk of developing BC. Although Sona’s study suggested a potential reduction in BC risk among individuals with T1D, which was conducted 7 years ago, it may not reflect the most recent evidence [39]. In contrast, a more recent study by Xiong et al. indicated that T1D may be associated with an increased risk of BC [15]. To reassess this issue, we conducted an updated meta‐analysis, which also suggested a possible inverse association between T1D and BC risk, in line with Sona’s findings. In the five articles addressing this association, a pooled analysis encompassing five study populations reported estimates consistent with an inverse association, three showed no statistically significant association, and one suggested an increased risk. This latter study was the only prospective cohort study among the nine. Although the authors of the pooled analysis suggested that their contradictory results might be influenced by other BC risk factors (e.g., number of pregnancies), the underlying mechanisms remain unclear, necessitating further research [14]. However, the link between T1D and molecular subtypes of BC, particularly regarding ER expression status, has not been investigated in prior studies. To address this, our MR analysis provided evidence consistent with an inverse association between genetically predicted T1D and ER‐positive BC risk, whereas no statistically significant association was observed for ER‐negative BC. The findings emphasize the importance of further investigation into the link between T1D and specific BC subtypes, with particular focus on ER status. Moderate heterogeneity was observed in the meta‐analysis (*I* ^2^ = 54%), suggesting variability among included studies. This heterogeneity likely arises from several methodological and population‐related factors. First, differences in study design—particularly between retrospective and prospective cohorts—may have contributed to variability in effect estimates. Retrospective studies are more susceptible to misclassification bias and incomplete confounder adjustment compared with prospective designs. Second, criteria for defining T1D were not consistent across studies. While most studies relied on ICD codes, others used age at diagnosis or insulin dependence as a proxy, which could lead to the inadvertent inclusion of individuals with T2D. Such misclassification would attenuate the true association and introduce between‐study variability. Third, differences in follow‐up duration, calendar period, and regional healthcare systems may have influenced both diabetes diagnosis accuracy and cancer ascertainment. Fourth, the distribution of potential confounders such as BMI, reproductive history, and exogenous insulin use differed across populations, which could influence the observed association. Although the observed heterogeneity was moderate, the direction of effect was consistent across most studies, supporting the robustness of the pooled estimate. Sensitivity analyses confirmed that excluding any single study did not materially alter the summary effect size. Moreover, the MR analysis, which is less susceptible to conventional environmental confounding and reverse causation, yielded findings broadly consistent with the meta‐analysis, providing complementary genetic evidence despite heterogeneity in the observational findings. Nevertheless, caution is warranted when interpreting the pooled risk estimates, and future large‐scale studies using standardized definitions of T1D and harmonized data collection are needed to minimize heterogeneity and improve comparability.

Although the inverse associations observed in both the meta‐analysis and MR analyses reached statistical significance, the magnitude of the effect was modest. The pooled relative effect estimate from the observational meta‐analysis was 0.91, while the IVW estimates for overall and ER‐positive BC were 0.957 and 0.953, respectively. Therefore, statistical significance should not be interpreted as indicating a large or necessarily clinically meaningful reduction in BC risk at the individual level. This consideration is particularly relevant to analyses based on large GWAS datasets, in which relatively small effect estimates may achieve statistical significance. Accordingly, the present findings should primarily be interpreted as evidence regarding the direction and potential biological relevance of the association rather than as evidence of a substantial reduction in individual BC risk.

The pleiotropy analyses also warrant careful interpretation. Although the MR‐PRESSO global test indicated evidence of horizontal pleiotropy for overall, ER‐positive, and ER‐negative BC, the MR‐Egger intercept tests were not statistically significant. These findings are not necessarily contradictory because MR‐PRESSO is particularly sensitive to heterogeneous or outlying SNP‐specific effects, whereas the MR‐Egger intercept assesses whether pleiotropic effects are directional on average across the genetic instruments [34, 35]. The observed pattern may therefore reflect balanced pleiotropy or pleiotropic effects driven by a limited number of outlier variants rather than systematic directional bias. Importantly, removal of MR‐PRESSO‐identified outliers did not materially alter the effect estimates, as indicated by the nonsignificant distortion tests. Together with the consistency between the IVW and weighted‐median estimates and the stability observed in leave‐one‐out analyses, these findings suggest that the detected pleiotropy was unlikely to fully explain the observed inverse associations. Nevertheless, residual horizontal pleiotropy cannot be completely excluded, and the MR results should therefore be interpreted as supporting, rather than definitively establishing, a potential causal relationship between T1D and BC risk.

Several biological pathways may potentially contribute to the observed inverse association between T1D and BC, although these mechanisms remain speculative and cannot be established by the present study. One possible explanation involves alterations in sex hormone bioavailability. Most circulating estrogen is protein‐bound, including binding to SHBG, whereas only a small fraction circulates in an unbound and biologically active form [40]. Individuals with T1D may exhibit altered SHBG concentrations, potentially reflecting changes in hepatic insulin exposure and metabolic regulation [41–43]. Higher SHBG concentrations could reduce the bioavailability of circulating estrogens and may therefore be particularly relevant to ER‐positive breast carcinogenesis. This hypothesis could provide one possible biological explanation for the stronger inverse association observed for ER‐positive than for ER‐negative BC in our MR analysis. However, because SHBG and estrogen concentrations were not directly evaluated in the present study, this interpretation remains indirect and requires further mechanistic validation.

Alterations in insulin and IGF‐1 signaling represent another potential pathway. T1D is characterized by autoimmune destruction of pancreatic β‐cells and loss of endogenous insulin secretion, resulting in relative intraportal hypoinsulinemia. Portal insulin delivery is an important regulator of hepatic GH sensitivity and IGF‐1 production, and reduced circulating IGF‐1 concentrations have frequently been observed in individuals with T1D, particularly in the presence of suboptimal metabolic control [20, 44, 45]. Insulin and IGF‐1 can activate downstream signaling networks, including the PI3K/AKT/mTOR and MAPK pathways, which regulate cellular proliferation, survival, metabolism, and tumor progression [42, 46]. Therefore, alterations in insulin/IGF‐1 signaling could theoretically modify proliferative signaling in breast tissue.

However, the metabolic environment in T1D is considerably more complex than a state of simple insulin deficiency. Individuals with T1D generally require lifelong exogenous insulin replacement, and conventional subcutaneous insulin administration does not reproduce the physiological portal‐to‐systemic insulin gradient. Consequently, peripheral insulin exposure may be relatively increased while hepatic portal insulinization remains lower than that produced by endogenous pancreatic secretion. The route, dose, intensity, and duration of insulin treatment, as well as glycemic control, may therefore modify the GH/IGF‐1 axis and other metabolic pathways over the course of T1D [45, 47]. More recent evidence further emphasizes that intraportal insulin is an important determinant of hepatic GH sensitivity, IGF‐1 production, and IGF‐binding protein regulation [44]. Accordingly, the relationship between insulin exposure and BC risk in T1D should not be interpreted simply as a consequence of reduced endogenous insulin production. The net effect is likely to vary according to insulin regimen, metabolic control, residual β‐cell function, disease duration, and individual metabolic characteristics.

Immune and inflammatory mechanisms may also be relevant. T1D is fundamentally an autoimmune disease characterized by immune‐mediated destruction of pancreatic β‐cells and persistent abnormalities in immune regulation. Alterations in inflammatory and regulatory immune pathways may theoretically influence cancer immune surveillance and the tumor microenvironment. Moreover, the IGF system itself has been implicated in the regulation of inflammatory and immune‐cell function in T1D [20]. However, direct evidence demonstrating that T1D‐specific autoimmunity protects against breast carcinogenesis remains limited. Thus, alterations in sex hormone bioavailability, insulin/IGF‐1 signaling, metabolic regulation, and immune function should be regarded as biologically plausible hypotheses rather than established mechanisms underlying the observed association.

Several clinically relevant characteristics of T1D could not be evaluated in the present study and may modify the observed association. In particular, diabetes duration, age at T1D onset, long‐term glycemic control as reflected by glycated hemoglobin (HbA1c), insulin treatment intensity and regimen, and the presence of chronic diabetic complications were not consistently available across the observational studies or summary‐level GWAS datasets. These factors may influence cumulative exposure to hyperglycemia, exogenous insulin, inflammation, and endocrine alterations and could therefore modify BC risk over time. For example, individuals with long‐standing T1D and persistently poor glycemic control may have a substantially different metabolic and inflammatory environment from those with shorter disease duration and well‐controlled disease. The abnormalities of the GH/IGF‐1 axis in T1D are themselves influenced by metabolic control and insulin delivery [45, 47]. Consequently, the observed association should not be assumed to be uniform across all individuals with T1D. Future longitudinal studies incorporating detailed information on disease duration, HbA1c, insulin treatment, residual β‐cell function, and diabetes‐related complications will be important for determining whether these clinical characteristics modify the association between T1D and BC.

The stronger inverse association observed for ER‐positive BC also raises the possibility that the relationship between T1D and BC differs according to hormonal and molecular context. Future studies should evaluate this association according to menopausal status and age at BC diagnosis and, where sufficiently detailed outcome data are available, across more refined molecular subtypes, including luminal A, luminal B, HER2‐enriched, and triple‐negative BC. Stratification according to age at T1D onset and diabetes duration may provide additional insight into whether cumulative metabolic, hormonal, or inflammatory exposures contribute to subtype‐specific associations. Such analyses may help determine whether the observed ER‐specific pattern reflects differences in estrogen‐related pathways or other biological mechanisms.

Several limitations of this study should be acknowledged. First, the definition of T1D varied across the included observational studies. Although some studies identified T1D using diagnostic codes, others relied on age at diabetes diagnosis or insulin dependence as proxies, which may have resulted in misclassification of some individuals with T2D. Notably, the significant subgroup difference according to the definition of T1D suggests that variation in disease classification may have contributed to the observed heterogeneity. Second, although subgroup analyses were performed to explore potential sources of heterogeneity, the relatively small number of included cohorts restricted statistical power, and some potentially important sources of heterogeneity could not be examined reliably through further stratification or meta‐regression. Third, the systematic review protocol was not prospectively registered before the review was initiated. Prospective registration can improve methodological transparency by prespecifying eligibility criteria, outcomes, and analytical strategies and thereby reducing concerns regarding selective methodological or outcome reporting. Although the literature search, eligibility criteria, data extraction procedures, quality assessment, and statistical methods were explicitly defined and are reported in detail in the present study, the absence of prospective registration remains a limitation and should be considered when interpreting the meta‐analytic findings. Future systematic reviews addressing this topic should prospectively register their protocols before study initiation. Fourth, detailed individual‐level clinical variables, including diabetes duration, age at T1D onset, long‐term HbA1c, insulin regimen and treatment intensity, chronic diabetic complications, reproductive factors, and menopausal status, were unavailable or inconsistently reported and therefore could not be adequately evaluated. Residual confounding by these factors may therefore remain in the observational evidence. Fifth, the GWAS datasets and most observational cohorts predominantly included individuals of European ancestry, which may limit the generalizability of the findings to other ancestral populations. Although the use of relatively homogeneous ancestry in the MR analysis may reduce population stratification, residual population structure cannot be completely excluded. Sixth, the available genetic outcome data allowed stratification by ER status but not by HER2 status or more refined molecular subtypes such as luminal A, luminal B, HER2‐enriched, and triple‐negative BC. In addition, although the MR‐PRESSO distortion tests and leave‐one‐out analyses suggested that the principal estimates were not driven by individual outlier variants, evidence of horizontal pleiotropy was detected by MR‐PRESSO. Residual pleiotropy therefore cannot be entirely excluded. More broadly, MR relies on assumptions regarding instrument relevance, independence, and exclusion restriction that cannot be fully verified empirically, and violations of these assumptions may bias causal estimates [32]. Genetic instruments are also generally interpreted as representing lifelong differences in genetic liability and may not directly reproduce the biological consequences of clinically diagnosed T1D, varying disease duration, glycemic control, or changes in insulin therapy over time. Finally, the observed effect sizes were modest. Therefore, the statistically significant inverse associations should not be interpreted as indicating a large protective effect at the individual level. These limitations underscore the need for larger multiancestry studies with detailed clinical phenotyping and complementary mechanistic investigations.

## 5. Conclusion

Our findings indicate that the combined observational and genetic evidence is consistent with a possible inverse association between T1D and BC risk, particularly for ER‐positive tumors, whereas no significant association was observed for ER‐negative BC. These findings should be interpreted cautiously and warrant further investigation in larger and more diverse populations to confirm the observed associations and clarify the underlying biological mechanisms.

## Author Contributions

Bo Xu conceived and designed the study, supervised the research process, and critically revised the manuscript for important intellectual content. Yi Xie conducted the meta‐analysis and Mendelian randomization analyses; performed data collection, management, and statistical analyses using relevant software; interpreted the results; prepared the figures and initial tables; and drafted the manuscript. Jianhua Guan and Junda Li contributed to the meta‐analysis, including literature screening, data extraction, and data verification. Qimou Lin and Junhong Wen contributed to the Mendelian randomization analysis, including data processing, methodological assessment, and interpretation of the findings. Lin Yuan contributed to the preparation, visualization, and refinement of figures and tables. All authors participated in the discussion and interpretation of the results and critically reviewed the manuscript,

## Funding

This research received no external funding.

## Disclosure

All authors approved the final version for publication and agreed to be accountable for all aspects of the work.

## Conflicts of Interest

The authors declare no conflicts of interest.

## Supporting Information

Additional supporting information can be found online in the Supporting Information section.

## Supporting information


**Supporting Information** This manuscript is accompanied by Supporting Information that provide additional details on the data sources, quality assessment, and analytical procedures used in the meta‐analysis and Mendelian randomization (MR) analyses. Supporting Table S1 summarizes the characteristics of the GWAS datasets used in the MR analysis. Supporting Table S2 presents the Newcastle–Ottawa Scale (NOS) assessment of the observational studies included in the meta‐analysis. Supporting Table S3 details the characteristics and strength of the genetic instrumental variables for T1D used in the forward MR analysis. Supporting Tables S4–S6 report the genetic associations of the selected instrumental variables with BC (Table S4), ER‐positive BC (Table S5), and ER‐negative BC (Table S6), respectively, including the effect and other alleles, *p* values, beta coefficients, and standard errors for each SNP. Supporting R Code provides the R scripts used for the meta‐analysis and the Mendelian randomization analyses.

## Data Availability

The data used in this study are publicly available. Data included in the meta‐analysis were extracted from previously published studies, all of which are cited in the article and its Supporting Information. Summary‐level GWAS data used for the Mendelian randomization analyses were obtained from publicly accessible sources, including the IEU OpenGWAS database and the FinnGen consortium. Further information regarding the datasets analyzed during the current study is available from the corresponding author upon reasonable request. The R code used for the meta‐analysis and Mendelian randomization analyses is available in the Supporting Information.
