# Supplementary material for: Type 1 Diabetes and Breast Cancer Risk: Evidence From an Updated Meta‐Analysis and Mendelian Randomization Study
Source: Int J Endocrinol. 2026 Nov 5;2026:2837214. doi: 10.1155/ije/2837214 (PMC13740488; doi:10.1155/ije/2837214)
Supplement: Supplementary file 1 [file IJE-2026-2837214-s001.zip › Supplementary R Code.docx]

**Supplementary Material: R scripts used for the meta-analysis and Mendelian randomization analyses.**

# **1. R scripts used for the meta-analysis**

library(meta)

data$lnHR<- log(data$HR)

data$selnHR <- (log(data$UCL)-log(data$LCI))/(2*1.96)

MetaHR<-metagen(lnHR,

selnHR,

sm="HR",

data=data,

studlab=paste(data$Study),

fixed=F,

random=T,

backtransf=T)

settings.meta("JAMA")

settings.meta("Revman5")

forest(MetaHR)

summary(MetaHR)

funnel(MetaHR)

cc <- funnel(MetaHR,

conbed.fixed = TRUE,

level = 0.95,

contour=c(0.9,0.95,0.99),

lwd = 2,

cex=2,

pch=16,

studlab=TRUE,

cex.stulab=1.25

)$col.contour

cc <- funnel(MetaHR,

random.fixed = TRUE,

level = 0.95,

contour=c(0.9,0.95,0.99),

)$col.contour

legend(

x=1.6,

y=0.01,

legend=c("p<0.01"),

fill = cc)

title("Contour-Enhanced Funnel Plot")

begg_test <- metabias(MetaHR,method="begg")

funnel(begg_test)

begg_plot <- ggplot(data,aes(x =sei,y=yi))+geom_point(color="blue")+geom_smooth(method = "lm",se=FALSE,color="red")+labs(title = "Begg's Test:Funnel Plot",x="Standard Error(sei)",y="Effec Size(yi)")+theme_minimal()

print(begg_plot)

metabias(MetaHR,method="linreg")

mytrimfill<-trimfill(MetaHR)

summary(mytrimfill)

funnel(mytrimfill,

conbed.fixed = TRUE,

level = 0.95,

contour=c(0.9,0.95,0.99),)

title("Funnel Plot (Trim & Fill Method)")

summary(MetaHR)

metainf(MetaHR,pooled = "random")

forest(metainf(MetaHR,pooled = "random"))

dat$Effect_size <- factor(

dat$`Effect size`,

levels = c("HR", "SIR")

)

dat$T1D_definition <- factor(

dat$`Definition of T1D`,

levels = c("age", "ICD")

)

dat$Publication_source <- factor(

dat$`Publication source`,

levels = c("Carstensen study", "other study")

)

dat$BC_cases <- gsub("＞", ">", dat$`BC cases among T1D patients`)

dat$BC_cases <- gsub("＜", "<", dat$BC_cases)

dat$BC_cases <- factor(

dat$BC_cases,

levels = c("<100", ">100")

)

dat$Person_years <- gsub("＞", ">", dat$`Person-years at risk`)

dat$Person_years <- gsub("＜", "<", dat$Person_years)

dat$Person_years <- factor(

dat$Person_years,

levels = c("<200000", ">200000")

)

table(dat$Effect_size, useNA = "ifany")

table(dat$T1D_definition, useNA = "ifany")

table(dat$Publication_source, useNA = "ifany")

table(dat$BC_cases, useNA = "ifany")

table(dat$Person_years, useNA = "ifany")

subgroup_meta <- function(data, subgroup){

d <- data[!is.na(data[[subgroup]]), ]

groups <- levels(droplevels(d[[subgroup]]))

results <- list()

for(g in groups){

dg <- d[d[[subgroup]] == g, ]

if(nrow(dg) >= 2){

fit <- rma(

yi = yi,

vi = vi,

data = dg,

method = "REML"

)

results[[g]] <- data.frame(

Variable = subgroup,

Subgroup = g,

Studies = nrow(dg),

Effect = exp(fit$b[1]),

LCI = exp(fit$ci.lb),

UCI = exp(fit$ci.ub),

P_value = fit$pval,

I2 = fit$I2,

Q_p = fit$QEp

)

} else {

results[[g]] <- data.frame(

Variable = subgroup,

Subgroup = g,

Studies = nrow(dg),

Effect = NA,

LCI = NA,

UCI = NA,

P_value = NA,

I2 = NA,

Q_p = NA

)

}

}

subgroup_results <- do.call(rbind, results)

formula_mod <- as.formula(

paste0("~ ", subgroup)

)

mod_fit <- rma(

yi = yi,

vi = vi,

mods = formula_mod,

data = d,

method = "REML"

)

P_interaction <- mod_fit$QMp

subgroup_results$P_interaction <- NA

subgroup_results$P_interaction[1] <- P_interaction

return(subgroup_results)

}

res_effect <- subgroup_meta(

dat,

"Effect_size"

)

res_effect

res_definition <- subgroup_meta(

dat,

"T1D_definition"

)

res_definition

res_source <- subgroup_meta(

dat,

"Publication_source"

)

res_source

res_BCcases <- subgroup_meta(

dat,

"BC_cases"

)

res_BCcases

res_personyears <- subgroup_meta(

dat,

"Person_years"

)

res_personyears

subgroup_results <- bind_rows(

res_effect,

res_definition,

res_source,

res_BCcases,

res_personyears

)

subgroup_results

subgroup_results_final <- subgroup_results %>%

mutate(

Effect = round(Effect, 3),

LCI = round(LCI, 3),

UCI = round(UCI, 3),

I2 = round(I2, 1),

P_value = signif(P_value, 3),

Q_p = signif(Q_p, 3),

P_interaction = signif(P_interaction, 3),

`Effect (95% CI)` =

paste0(

Effect,

" (",

LCI,

"–",

UCI,

")"

)

) %>%

select(

Variable,

Subgroup,

Studies,

`Effect (95% CI)`,

P_value,

I2,

Q_p,

P_interaction

)

subgroup_results_final

write_xlsx(

subgroup_results_final,

"Subgroup_analysis_results.xlsx"

)

# **2. R scripts used for the Mendelian randomization analyses**

library(ieugwasr)

library(TwoSampleMR)

library(MRInstruments)

T1DM_exp_dat<-extract_instruments(outcomes = "ebi-a-GCST90018925")

write.csv(T1DM_exp_dat,"T1DM_exp_dat.csv")

library(vroom)

library(tidyr)

library(plyr)

library(data.table)

breast_cancer<-vroom('BC.gz', col_names = TRUE)

View(breast_cancer)

write.csv(breast_cancer,"breast_cancer.csv")

c<-merge(T1DM_exp_dat,breast_cancer,by.x = "SNP",by.y = "rsids")

write.csv(c,"outcome.csv")

outcome_dat <- read_outcome_data(snps = T1DM_exp_dat$SNP, filename = "outcome.csv", sep = ",", snp_col ="SNP",beta_col ="beta",se_col ="se",effect_allele_col ="effect_allele",other_allele_col ="other_allele",pval_col ="p")

dat<-harmonise_data(exposure_dat = T1DM_exp_dat,outcome_dat = outcome_dat)

mr(dat)

generate_odds_ratios(mr_res = mr(dat,method_list = c("mr_ivw","mr_weighted_median","mr_egger_regression")))

mr(dat,method_list = c("mr_ivw","mr_weighted_median","mr_egger_regression"))

mr_scatter_plot(mr_results = mr(dat,method_list = c("mr_ivw","mr_weighted_median","mr_egger_regression")),dat)

mr_heterogeneity(dat)

run_mr_presso(dat,NbDistribution = 3000)

mr_funnel_plot(singlesnp_results = mr_singlesnp(dat))

mr_forest_plot(singlesnp_results = mr_singlesnp(dat))

mr_pleiotropy_test(dat)

mr_leaveoneout(dat)

generate_odds_ratios(mr_res = mr_leaveoneout(dat))

mr_leaveoneout_plot(leaveoneout_results = mr_leaveoneout(dat))

generate_odds_ratios(mr_leaveoneout_plot(leaveoneout_results = mr_leaveoneout(dat)))

generate_odds_ratios(leaveoneout_results = mr_leaveoneout(dat))

mr_leaveoneout_plot(generate_odds_ratios(mr_res = mr_leaveoneout(dat)))

mr_leaveoneout_plot(generate_odds_ratios(mr_res = mr_leaveoneout(dat)))
