# Supplementary material for: Type 1 Diabetes and Breast Cancer Risk: Evidence From an Updated Meta‐Analysis and Mendelian Randomization Study
Source: Int J Endocrinol. 2026 Nov 5;2026:2837214. doi: 10.1155/ije/2837214 (PMC13740488; doi:10.1155/ije/2837214)
Supplement: Supplementary file 1 [file IJE-2026-2837214-s001.zip › STROBE-MR-checklist.docx]

**STROBE-MR checklist of recommended items to address in reports of Mendelian randomization studies**^1^ ^2^

| **Item No.** | **Section** | **Checklist item** | **Page No.** | **Relevant text from manuscript** |
| --- | --- | --- | --- | --- |
| 1 | **TITLE and ABSTRACT** | Indicate Mendelian randomization (MR) as the study’s design in the title and/or the abstract if that is a main purpose of the study | 1 | Association Between Type 1 Diabetes and Breast Cancer Risk: A Combined Observational and Mendelian Randomization Study |
|  | **INTRODUCTION** |  |  |  |
| 2 | **Background** | Explain the scientific background and rationale for the reported study. What is the exposure? Is a potential causal relationship between exposure and outcome plausible? Justify why MR is a helpful method to address the study question | 2 | Extensive observational evidence has sought to clarify whether T1D influences BC risk; nonetheless, the current literature provides conflicting and inconclusive results. As early as 1997, Hjaldgrim et al. reported no statistically significant association between T1D and BC risk, with similar conclusions drawn by Shu et al., Hsu et al., and Swerdlow et al. in later studie. In contrast, Carstensen et al.’s extensive multinational cohort study, spanning five countries, suggested a potential protective effect of T1D against BC, whereas Xiong et al. reported an increased risk in women with T1D compared to non-diabetic controls. Such inconsistent evidence highlights the importance of reassessing this relationship with more recent data and robust analytical methods.  Given these inconsistencies and the potential biological mechanisms linking T1D and BC, further investigation is warranted. Therefore, by pooling data from prior observational studies, this study offers a comprehensive meta-analytic evaluation of the T1D–BC association. Complementing this, a Mendelian randomization (MR) approach is applied, utilizing T1D-associated genetic variants as instruments to reinforce causal conclusions and limit residual confounding. By integrating both meta-analytic and MR approaches, this study strives to present robust and multifaceted insights into the T1D–BC relationship, yielding new understanding of potential causal pathways and implications for clinical practice. |
| 3 | **Objectives** | State specific objectives clearly, including pre-specified causal hypotheses (if any). State that MR is a method that, under specific assumptions, intends to estimate causal effects | 2 | Therefore, by pooling data from prior observational studies, this study offers a comprehensive meta-analytic evaluation of the T1D–BC association. Complementing this, a Mendelian randomization (MR) approach is applied, utilizing T1D-associated genetic variants as instruments to reinforce causal conclusions and limit residual confounding. |
|  | **METHODS** |  |  |  |
| 4 | **Study design and data sources** | Present key elements of the study design early in the article. Consider including a table listing sources of data for all phases of the study. For each data source contributing to the analysis, describe the following: |  |  |
|  | a) | Setting: Describe the study design and the underlying population, if possible. Describe the setting, locations, and relevant dates, including periods of recruitment, exposure, follow-up, and data collection, when available. | 4 | The study population of the original GWAS comprised individuals of European descent. Exposure data for T1D originated from the IEU Open GWAS database (GWAS ID: ebi-a-GCST90018925). Outcomes data for BC—including total cases, estrogen receptor (ER)-positive and ER-negative subtypes—were retrieved from FinnGen (R12). |
|  | b) | Participants: Give the eligibility criteria, and the sources and methods of selection of participants. Report the sample size, and whether any power or sample size calculations were carried out prior to the main analysis | 4 | The study population of the original GWAS comprised individuals of European descent. Exposure data for T1D were obtained from the IEU Open GWAS database (GWAS ID: ebi-a-GCST90018925), which was selected based on its large sample size, recent availability, and greater number of genetic variants available for instrument selection[23]. Outcome data for overall BC, ER-positive BC, and ER-negative BC were derived from FinnGen (R12), an independent cohort chosen to minimize potential sample overlap with the exposure dataset. Detailed characteristics of all GWAS datasets are presented in Table S1. |
|  | c) | Describe measurement, quality control and selection of genetic variants | 4 | At the preliminary stage, we extracted SNPs that exhibited genome-wide significant associations (P < 5 × 10⁻⁸) with the exposure trait. Linkage disequilibrium clumping (R² < 0.001; distance > 10 Mb) was subsequently performed to ensure SNP independence.  SNPs absent in the outcomes dataset and palindrome SNPs were excluded to mitigate bias.  “F-statistics were employed to detect weak instrument bias. |
|  | d) | For each exposure, outcome, and other relevant variables, describe methods of assessment and diagnostic criteria for diseases | 4 | Exposure data for T1D originated from the IEU Open GWAS database.”  Outcomes data for BC—including total cases, estrogen receptor (ER)-positive and ER-negative subtypes—were retrieved from FinnGen (R12). |
|  | e) | Provide details of ethics committee approval and participant informed consent, if relevant |  | NA |
| 5 | **Assumptions** | Explicitly state the three core IV assumptions for the main analysis (relevance, independence and exclusion restriction) as well assumptions for any additional or sensitivity analysis | 3 | The MR analysis is built up on three essential assumptions: single nucleotide polymorphisms (SNPs) must have a robust association with T1D (the relevance criterion); they must be unrelated to confounding factors (the independence criterion); and their influence on breast cancer must be mediated exclusively via T1D (the exclusion restriction criterion). |
| 6 | **Statistical methods: main analysis** | Describe statistical methods and statistics used |  |  |
|  | a) | Describe how quantitative variables were handled in the analyses (i.e., scale, units, model) | 4 | The analysis uses summary-level data (HRs and 95% CIs). Three MR approaches—inverse variance weighted (IVW), weighted median, and MR-Egger—were applied to quantify causal associations. |
|  | b) | Describe how genetic variants were handled in the analyses and, if applicable, how their weights were selected | 4 | At the preliminary stage, we extracted SNPs that exhibited genome-wide significant associations (P < 5 × 10⁻⁸  Linkage disequilibrium clumping (R² < 0.001; distance > 10 Mb) was subsequently performed. |
|  | c) | Describe the MR estimator (e.g. two-stage least squares, Wald ratio) and related statistics. Detail the included covariates and, in case of two-sample MR, whether the same covariate set was used for adjustment in the two samples | 4 | Three MR approaches—inverse variance weighted (IVW), weighted median, and MR-Egger—were applied to quantify causal associations. |
|  | d) | Explain how missing data were addressed | 4 | SNPs absent in the outcomes dataset and palindrome SNPs were excluded to mitigate bias. |
|  | e) | If applicable, indicate how multiple testing was addressed |  | NA |
| 7 | **Assessment of assumptions** | Describe any methods or prior knowledge used to assess the assumptions or justify their validity | 4 | Heterogeneity among genetic instruments was evaluated using Cochran’s Q test.”  Horizontal pleiotropy was investigated through MR-Egger regression and MR-PRESSO. |
| 8 | **Sensitivity analyses and additional analyses** | Describe any sensitivity analyses or additional analyses performed (e.g. comparison of effect estimates from different approaches, independent replication, bias analytic techniques, validation of instruments, simulations) | 4 | Heterogeneity among genetic instruments was evaluated using Cochran’s Q test, where p-values exceeding 0.05 suggested its absence. Horizontal pleiotropy was investigated through MR-Egger regression and MR-PRESSO, with a zero intercept (p > 0.05) indicating no evidence of pleiotropy. Leave-one-out sensitivity analyses verified the stability of the results, ensuring that no single SNP disproportionately influenced the overall causal estimate. |
| 9 | **Software and pre-registration** |  |  |  |
|  | a) | Name statistical software and package(s), including version and settings used | 4 | R software (version 4.3.1) was used to perform all statistical analyses, utilizing the packages ‘TwoSampleMR’ (0.6.3), ‘MRPRESSO’ (1.0), and ‘MendelianRandomization’ (0.3.2). |
|  | b) | State whether the study protocol and details were pre-registered (as well as when and where) |  | Not reported |
|  | **RESULTS** |  |  |  |
| 10 | **Descriptive data** |  |  |  |
|  | a) | Report the numbers of individuals at each stage of included studies and reasons for exclusion. Consider use of a flow diagram |  | A set of 18 SNPs with strong associations to T1D was selected to function as instrumental variables (IVs). The rs3842753 genetic locus was excluded due to its potential confounding association with body mass index (BMI), ensuring the robustness of IV selection. Consequently, 17 SNPs were included in the final analysis. Detailed information on the selected IVs is provided in Table S2. |
|  | b) | Report summary statistics for phenotypic exposure(s), outcome(s), and other relevant variables (e.g. means, SDs, proportions) |  | Detailed study-specific results are presented in Figure 7 , Table 2 and Table S3.  Detailed study-specific results are presented in Figure 8 , Table 2 and Table S4.  Detailed study-specific results are presented in Figure 9 , Table 2 and Table S5. |
|  | c) | If the data sources include meta-analyses of previous studies, provide the assessments of heterogeneity across these studies |  | NA |
|  | d) | For two-sample MR:  i.  Provide justification of the similarity of the genetic variant-exposure associations between the exposure and outcome samples  ii.  Provide information on the number of individuals who overlap between the exposure and outcome studies | 4 | The study population of the original GWAS comprised individuals of European descent.  F-statistics were employed to detect weak instrument bias and sample overlap.  an independent cohort chosen to minimize potential sample overlap with the exposure dataset. |
| 11 | **Main results** |  |  |  |
|  | a) | Report the associations between genetic variant and exposure, and between genetic variant and outcome, preferably on an interpretable scale | 4,5,6 | A set of 18 SNPs with strong associations to T1D was selected to function as instrumental variables (IVs). The rs3842753 genetic locus was excluded due to its potential confounding association with body mass index (BMI), ensuring the robustness of IV selection. Consequently, 17 SNPs were included in the final analysis. Detailed information on the selected IVs is provided in Table S2.  Detailed study-specific results are presented in Figure 7 , Table 2 and Table S3.  Detailed study-specific results are presented in Figure 8 , Table 2 and Table S4.  Detailed study-specific results are presented in Figure 9 & Table 2. |
|  | b) | Report MR estimates of the relationship between exposure and outcome, and the measures of uncertainty from the MR analysis, on an interpretable scale, such as odds ratio or relative risk per SD difference | 4,5,6 | A significant protective effect of T1D on BC was observed through both IVW and Weighted Median methods (IVW: HR = 0.957, 95% CI: 0.919–0.997, P = 0.033; Weighted Median: HR = 0.959, 95% CI: 0.935–0.983, P = 0.001). However, the MR-Egger analysis yielded more conservative and statistically non-significant results (HR = 0.979, 95% CI: 0.915–1.047, P = 0.542). Detailed study-specific results are presented in Figure 7 , Table 2 and Table S3.  The IVW and Weighted Median analyses consistently demonstrated that T1D was significantly associated with a lower risk of ER-positive BC (IVW: HR = 0.953, 95% CI: 0.918–0.990, P = 0.013; Weighted Median: HR = 0.952, 95% CI: 0.925–0.981, P = 0.001). In contrast, MR-Egger analysis yielded more conservative and non-significant results (HR = 0.971, 95% CI: 0.912–1.034, P = 0.377). Detailed study-specific results are presented in Figure 8 , Table 2 and Table S4.  No significant causal relationship was observed between T1D and ER-negative BC risk across all methods (IVW: HR = 0.967, 95% CI: 0.921–1.014, P = 0.162; MR-Egger: HR = 0.997, 95% CI: 0.921–1.078, P = 0.934; Weighted Median: HR = 0.974, 95% CI: 0.940–1.010, P = 0.147). Detailed study-specific results are presented in Figure 9 & Table 2. |
|  | c) | If relevant, consider translating estimates of relative risk into absolute risk for a meaningful time period |  | Not reported in the manuscript. |
|  | d) | Consider plots to visualize results (e.g. forest plot, scatterplot of associations between genetic variants and outcome versus between genetic variants and exposure) | 5,6 | Detailed study-specific results are presented in Figure 7 , Table 2 and Table S3.  Detailed study-specific results are presented in Figure 8 , Table 2 and Table S4.  Detailed study-specific results are presented in Figure 9 & Table 2. |
| 12 | **Assessment of assumptions** |  |  |  |
|  | a) | Report the assessment of the validity of the assumptions | 5,6,7 | MR-PRESSO analysis identified significant horizontal pleiotropy (Global Test P < 0.001). After excluding outlier SNPs, the causal estimate strengthened (Outlier-corrected Causal Estimate = -0.038; SE = 0.014; P = 0.018) without evidence of distortion (Distortion Test P = 0.517). Based on the Egger regression intercept (−0.009; SE = 0.011; P = 0.422), there was no statistical evidence of horizontal pleiotropy affecting the results.  MR-PRESSO analysis identified significant horizontal pleiotropy (Global Test P = 0.001). After excluding outlier SNPs, the causal estimate became more pronounced (Outlier-corrected Causal Estimate = -0.043; SE = 0.014; P = 0.009), with results remaining consistent pre- and post-correction (Distortion Test P = 0.517). Egger’s regression intercept (Egger intercept =−0.008; SE = 0.011; P = 0.476) indicated no significant pleiotropy.  Significant horizontal pleiotropy was identified by the MR-PRESSO global test (P = 0.001), suggesting potential exposure-independent effects of several instrumental variables. After excluding outlier SNPs, the causal estimate was -0.028 (SE = 0.018; P = 0.146), showing a consistent but statistically nonsignificant association. The distortion test (P = 0.576) indicated that outlier removal did not materially alter the causal estimate, supporting the robustness of the findings. No directional pleiotropy was detected based on the MR-Egger intercept test (intercept = −0.013; SE = 0.013; P = 0.352), implying that overall bias due to pleiotropy was minimal. |
|  | b) | Report any additional statistics (e.g., assessments of heterogeneity across genetic variants, such as *I^2^*, Q statistic or E-value) | 6,7 | Heterogeneity tests revealed significant variability among IVs (Cochran’s Q for IVW = 74.728, P < 0.001; MR-Egger = 71.481, P < 0.001).  Heterogeneity testing demonstrated statistically significant differences (Cochran’s Q for IVW = 41.980, P < 0.001; MR-Egger = 40.538, P < 0.001).  Heterogeneity tests revealed significant variability among the instrumental variables (Cochran’s Q for IVW = 46.620, P < 0.001; MR-Egger = 43.913, P < 0.001), indicating potential heterogeneity across SNPs. |
| 13 | **Sensitivity analyses and additional analyses** |  |  |  |
|  | a) | Report any sensitivity analyses to assess the robustness of the main results to violations of the assumptions | 6,7 | Leave-one-out sensitivity analyses verified the stability of the results, with no single SNP exerting a notable impact on the outcomes. Detailed study-specific results are presented in Figure 7 & Table 2.  Robustness of the findings was additionally supported by the leave-one-out analysis. Detailed study-specific results are presented in Figure 8 & Table 2.  Leave-one-out tests conducted as part of sensitivity analyses further validated the consistency of the findings. Detailed study-specific results are presented in Figure 9 , Table 2 and Table S5. |
|  | b) | Report results from other sensitivity analyses or additional analyses | 5,6,7 | MR-PRESSO analysis identified significant horizontal pleiotropy (Global Test P < 0.001). After excluding outlier SNPs, the causal estimate strengthened (Outlier-corrected Causal Estimate = -0.038; SE = 0.014; P = 0.018) without evidence of distortion (Distortion Test P = 0.517). Based on the Egger regression intercept (−0.009; SE = 0.011; P = 0.422), there was no statistical evidence of horizontal pleiotropy affecting the results.  MR-PRESSO analysis identified significant horizontal pleiotropy (Global Test P = 0.001). After excluding outlier SNPs, the causal estimate became more pronounced (Outlier-corrected Causal Estimate = -0.043; SE = 0.014; P = 0.009), with results remaining consistent pre- and post-correction (Distortion Test P = 0.517). Egger’s regression intercept (Egger intercept =−0.008; SE = 0.011; P = 0.476) indicated no significant pleiotropy.  Significant horizontal pleiotropy was identified by the MR-PRESSO global test (P = 0.001), suggesting potential exposure-independent effects of several instrumental variables. After excluding outlier SNPs, the causal estimate was -0.028 (SE = 0.018; P = 0.146), showing a consistent but statistically nonsignificant association. The distortion test (P = 0.576) indicated that outlier removal did not materially alter the causal estimate, supporting the robustness of the findings. No directional pleiotropy was detected based on the MR-Egger intercept test (intercept = −0.013; SE = 0.013; P = 0.352), implying that overall bias due to pleiotropy was minimal. |
|  | c) | Report any assessment of direction of causal relationship (e.g., bidirectional MR) |  | Not reported in the manuscript. |
|  | d) | When relevant, report and compare with estimates from non-MR analyses | 6 | Collectively, findings suggest that T1D may reduce BC risk, which is consistent with the results of the meta-analysis. |
|  | e) | Consider additional plots to visualize results (e.g., leave-one-out analyses) | 5，6 | Detailed study-specific results are presented in Figure 7 , Table 2 and Table S3.  Detailed study-specific results are presented in Figure 8 , Table 2 and Table S4.  Detailed study-specific results are presented in Figure 9 & Table 2. |
|  | **DISCUSSION** |  |  |  |
| 14 | **Key results** | Summarize key results with reference to study objectives | 9 | Our study suggests that T1D may confer a protective effect against BC, particularly for ER-positive subtypes, whereas no significant association was found for ER-negative cases. |
| 15 | **Limitations** | Discuss limitations of the study, taking into account the validity of the IV assumptions, other sources of potential bias, and imprecision. Discuss both direction and magnitude of any potential bias and any efforts to address them | 7,8 | the GWAS data in this study and the majority of included meta-analytic studies pertain to individuals of European descent, which may reduce the external validity of the results for other racial or ethnic groups. Further studies are needed to verify whether these results apply to more diverse populations. Fifth, we only examined the association between T1D and ER status in BC, without analyzing HER-2 status; this aspect warrants further investigation. Sixth, the HR for T1D in relation to BC risk was relatively low, suggesting that the reduction in risk may be limited. Finally, MR analysis itself has inherent limitations: (1) single nucleotide polymorphisms (SNPs) are generally assumed to have lifelong effects, but their influence may vary depending on physiological conditions, environmental exposures, or interactions with other genetic variants. Temporal variations in genetic effects may also impact the robustness of causal inference; (2) although MR studies can infer causality, they cannot fully elucidate the molecular mechanisms through which T1D affects BC risk. |
| 16 | **Interpretation** |  |  |  |
|  | a) | Meaning: Give a cautious overall interpretation of results in the context of their limitations and in comparison with other studies | 7 | This study provided an in-depth assessment of the link between T1D and BC risk by integrating evidence from observational studies using a meta-analytic approach and incorporating Mendelian randomization analyses. The meta-analysis showed that women with T1D exhibited a notably lower likelihood of developing BC than those without diabetes, although moderate heterogeneity was observed across studies. Complementary MR analyses supported a potential causal relationship, indicating that genetically proxied T1D corresponds to a reduced risk of overall and ER-positive BC, whereas no similar association was detected for ER-negative BC. The consistency of these findings across multiple analytical frameworks reinforces the hypothesis that T1D may confer a protective influence on certain molecular subtypes of breast cancer.  Recent meta-analyses have largely focused on evaluating the association of type 2 diabetes or diabetes as a whole with the risk of developing breast cancer. Although Sona’s study suggested a potential reduction in BC risk among individuals with T1D, which was conducted seven years ago, it may not reflect the most recent evidence. In contrast, a more recent study by Xiong et al. indicated that T1D may be associated with an increased risk of BC. |
|  | b) | Mechanism: Discuss underlying biological mechanisms that could drive a potential causal relationship between the investigated exposure and the outcome, and whether the gene-environment equivalence assumption is reasonable. Use causal language carefully, clarifying that IV estimates may provide causal effects only under certain assumptions | 7 | What mechanisms might explain how T1D reduces the risk of BC? Typically, most estrogen in the bloodstream is bound to plasma proteins, with only about 1–2% remaining in a free stat[34]. Approximately 30–50% of estrogen binds to sex hormone-binding globulin (SHBG), and the biologically active form in the body is primarily free estrogen. Patients with well-controlled T1D often exhibit normal-to-high or elevated SHBG levels[35]. This may be attributed to insulin therapy, which, under normal insulin sensitivity, suppresses hepatic de novo lipogenesis and consequently enhances SHBG synthesis[35, 36]. In T1D, exogenously administered insulin increases systemic insulin concentrations while decreasing portal levels compared to endogenous secretion, potentially altering liver metabolism and leading to higher SHBG production[36, 37]. Elevated SHBG levels can lower circulating free estrogen concentrations, thereby potentially reducing the risk of hormone receptor–positive BC. Insulin and insulin-like growth factor 1 (IGF-1) have the potential to enhance breast cell proliferation, growth, and tumorigenic transformation via stimulation of the PI3K/AKT signaling cascade. [36, 38]. Insulin has been shown to promote reactive oxygen species (ROS) formation in vascular smooth muscle cells by regulating coenzyme II, with the nicotinamide adenine dinucleotide phosphate (NADPH) oxidase pathway serving as a key mediator[39]. Through this activation, insulin-stimulated Akt/p70S6K1 and ERK pathways are further amplified, ultimately resulting in increased production of vascular endothelial growth factor (VEGF)[39, 40]. Collectively, these pathways enhance breast cancer development, including tumor growth, local infiltration, and metastasis. In contrast, the pathophysiology of T1D involves autoimmune loss of β-cells, leading to insufficient insulin production. The consequent deficiency in insulin and lower IGF-1 levels may inhibit these signaling networks, reducing breast cell proliferation, malignant progression, and overall breast cancer risk. |
|  | c) | Clinical relevance: Discuss whether the results have clinical or public policy relevance, and to what extent they inform effect sizes of possible interventions | 9 | These results underscore the need for further investigation to clarify the underlying biological mechanisms. |
| 17 | **Generalizability** | Discuss the generalizability of the study results (a) to other populations, (b) across other exposure periods/timings, and (c) across other levels of exposure | 8 | The GWAS data in this study and the majority of included meta-analytic studies pertain to individuals of European descent, which may reduce the external validity of the results for other racial or ethnic groups. |
|  | **OTHER INFORMATION** |  |  |  |
| 18 | **Funding** | Describe sources of funding and the role of funders in the present study and, if applicable, sources of funding for the databases and original study or studies on which the present study is based | 9 | This research received no external funding. |
| 19 | **Data and data sharing** | Provide the data used to perform all analyses or report where and how the data can be accessed, and reference these sources in the article. Provide the statistical code needed to reproduce the results in the article, or report whether the code is publicly accessible and if so, where | 9 | Summary-level GWAS data used for the Mendelian randomization analyses were obtained from publicly accessible sources, including the IEU OpenGWAS database and the FinnGen consortium. The R code used for the meta-analysis and Mendelian randomization analyses is available in the Supplementary Materials. |
| 20 | **Conflicts of Interest** | All authors should declare all potential conflicts of interest | 9 | The authors declare no competing interests. |

This checklist is copyrighted by the Equator Network under the Creative Commons Attribution 3.0 Unported (CC BY 3.0) license.

1.	Skrivankova VW, Richmond RC, Woolf BAR, Yarmolinsky J, Davies NM, Swanson SA, et al. Strengthening the Reporting of Observational Studies in Epidemiology using Mendelian Randomization (STROBE-MR) Statement. JAMA. 2021;under review.

2.	Skrivankova VW, Richmond RC, Woolf BAR, Davies NM, Swanson SA, VanderWeele TJ, et al. Strengthening the Reporting of Observational Studies in Epidemiology using Mendelian Randomisation (STROBE-MR): Explanation and Elaboration. BMJ. 2021;375:n2233.
